# Immunological evaluation of the new stable ultrasound contrast agent LK565: a phase one clinical trial

**DOI:** 10.1186/1476-7120-2-16

**Published:** 2004-09-10

**Authors:** B Funke, HK Maerz, S Okorokow, S Polata, I Lehmann, U Sack, P Wild, T Geisler, RJ Zotz

**Affiliations:** 1 Department of Internal Medicine/Cardiology, Klinikum Schwalmstadt Schwalmstadt, Germany; 2 Heart Center Leipzig, University of Leipzig, Germany; 3 Institute of Clinical Immunology and Transfusion Medicine, University of Leipzig, Germany

## Abstract

**Background:**

Ultrasound contrast agents (UCAs) allow the enhancement of vascular definition, thereby providing more diagnostic information. LK565 is a new second-generation UCA based on synthetic polymers of aspartic acid which is eliminated from the blood stream via phagocytosis. LK565 forms very stable air-filled microspheres and is capable of repeated passage through the pulmonary capillary bed after peripheral intravenous injection. This characteristic allows examination of the cardiac function or extracardiac vessel abnormalities up to 15 minutes.

**Methods:**

A phase one clinical study was conducted on 15 healthy volunteers to identify the development of an undesirable immune response. Phagocytosis capacity, TNF-α secretion, and MHC class II upregulation of monocytes was monitored, as well as microsphere specific antibody development (IgM, IgG). Furthermore, the kinetics of the activation surface markers CD69, CD25, CD71, and CD11b on leukocytes were analyzed.

**Results:**

Due to LK565-metabolism the administration of the UCA led to saturation of phagocytes which was reversible after 24 hrs. Compared to positive controls neither significant TNF-α elevation, neither MHC class II and activation surface markers upregulation, nor specific antibody development was detectable.

**Conclusion:**

The administration of LK565 provides a comfortable duration of signal enhancement, esp. in echocardiography, without causing a major activation cascade or triggering an adaptive immune response. To minimize the risk of undesirable adverse events such as anaphylactoid reactions, immunological studies should be included in clinical trials for new UCAs. The use of LK565 as another new ultrasound contrast agent should be encouraged as a safe means to provide additional diagnostic information.

## Background

**Figure 1:**
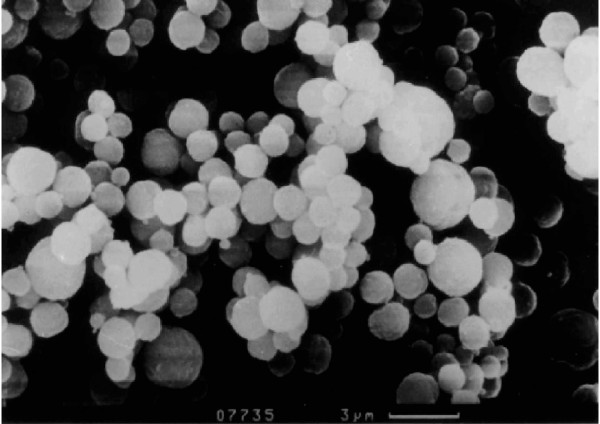
**Electron micrograph of LK565 **Electron micrograph of the particles of the new contrast agent LK565

**Figure 2:**
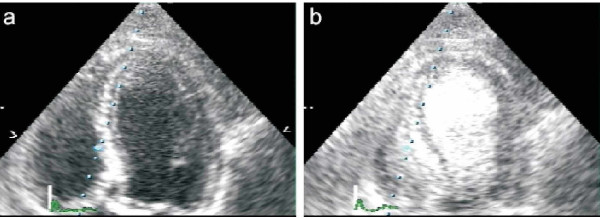
**Apical 4 chamber view with LK565 **Apical 4 chamber view before (a) and 1 minute after (b) intravenous LK565 application (4 mg/kg BW). Device: SONOS 5500, S3 Probe, harmonic

**Figure 3:**
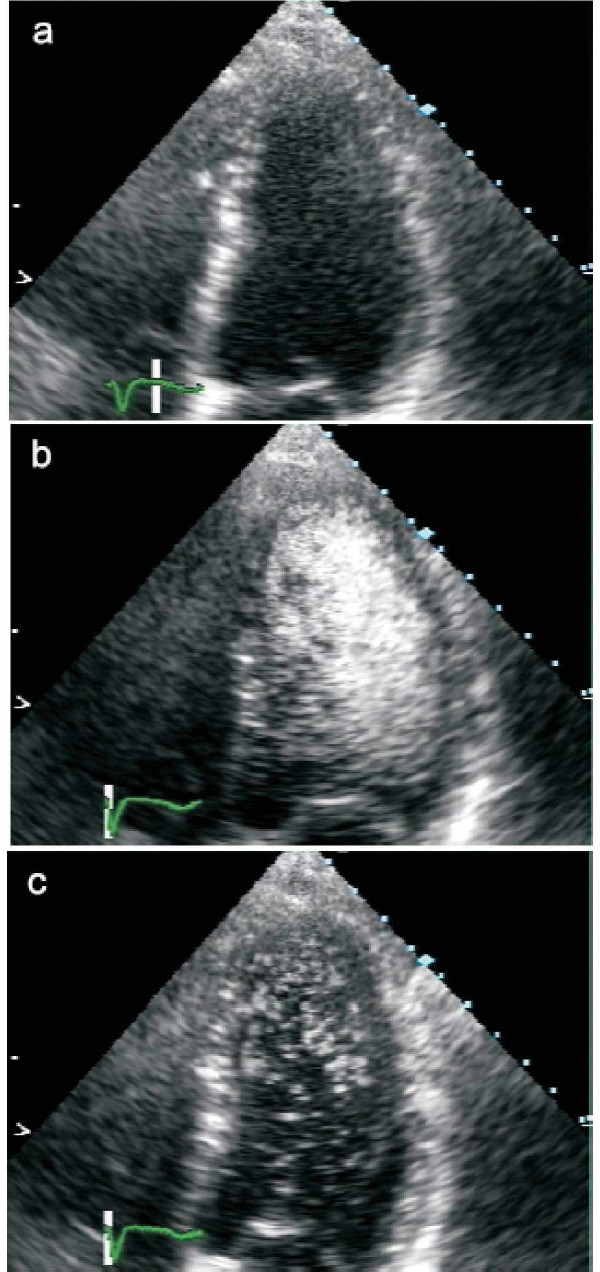
**Apical 4 chamber view with LK565 **Apical 4 chamber view before (a), 1 minute (b), and 30 minutes (c) after intravenous LK565 application (4 mg/kg BW) and intermittent ultrasound exposure. Device: SONOS 5500, S3 Probe, harmonic

Echocardiography allows the analysis of ventricular motion and heart-valve morphology. The discovery that injection of small air bubbles dispensed in saline are capable of causing an opacification of the heart [1,2] started an enormous effort to develop contrast agents to assist in diagnosing coronary artery disease and myocardial infarction [3]. Early developments led to stabilized bubbles in hyperosmolar solutions [4]. Albumin-stabilized bubbles were one of the first contrast agents to allow examination of both ventricles [5], but there are stability problems in patients with elevated pulmonary pressure [6]. Second-generation contrast agents improved the contrast especially in patients with subnormal echo signals [7-9]. LK565 and comparable substances represent a new concept in contrast agents as it consists of small, stable, air-filled particles [10] that provides a good contrast in both ventricles (figure 1, 2). Due to its high stability, LK565 improves examination of cardiac morphology and function as well as extracardiac vessel abnormalities for at least 15 minutes [11] (figure 3).

The particles consist of a synthetic polymer of aspartic acid. Via peptide bonds, the molecules form bold, stable beads filled with air. The average size of LK565 particles is 3 μm in diameter (figure 1). Because of their size and even polymeric surface the beads have similarities to microorganisms. This circumstance implies the induction of an immune response, which needs to be considered.

Results from previous studies indicate that phagocytes are responsible for eliminating the contrast agent from the blood stream [11]. In vitro, it has been shown that macrophages and neutrophils quickly devour the LK565 particles. Preliminary studies proved that the LK565 particles are phagocytosed by macrophages, resulting in slight activation with an increase in the intracellular calcium content. However, there were no symptoms of a respiratory burst [12].

The activation of macrophages can lead to different conditions: no activation, normal activation, and overreaction. In the last case, over-expression of tumor necrosis factor alpha (TNF-α) can lead to a septic shock by induction of intravascular blood clots apart from other effects, which may result in disseminated intravascular coagulation [13,14]. Furthermore, macrophages act as antigen-presenting cells which are highly important to induce an adaptive immune response, i.e. specific antibody production. It is probable that the LK565 particles attract antigen-presenting cells to present LK565 fragments as antigens. This may induce the production of specific antibodies against the contrast agent, which in rare cases could result in pathogenic immune reactions. The aim of this study was to examine a possible immune response to LK565 considering phagocytosis, TNF-α secretion by macrophages, lymphocyte activation and specific antibody production.

## Methods

### Volunteers

The experiment was approved by the local ethics committee (registration number 708/98). After medical examination, 15 healthy male volunteers under the age of 30 were enrolled in this phase one clinical study. Each volunteer was exposed to a single dose of the contrast agent LK565 applied intravenously. Four participants were exposed to the contrast agent LK565 for the second time because they had already been involved in a prior study [11]. The 15 volunteers were divided up into 3 groups (n = 5). The first group received 0.15 mg/kg bodyweight, the second group 0.4 mg/kg bodyweight and finally the third group 0.7 mg/kg LK565.

### Contrast agent

Prior to application, the contrast agent LK565 was dissolved in 10 ml physiological sodium chloride solution (37°C) under sterile conditions. To eliminate aggregations, the solution was filtered using a 20 μm mesh just before intravenous injection.

### Sampling

Heparinized blood samples (2 ml) were taken before application (t = 0), after 2 h, 6 h, 24 h, 48 h, 72 h, 96 h, and 1 ml after 10 d, 12 d and 14 d. For routine analysis, additional blood samples were drawn before and 2 hours after intravenous contrast agent injection. The volunteers were under medical care throughout the whole experiment.

### Phagocytosis

Phagocytosis capacity was determined in vitro before (t = 0) and after 2 h, 6 h and 24 h with Phagotest™ (Becton Dickinson, Germany) and analyzed via flow cytometry (FACScalibur™, BectonDickinson, Germany). FITC-labeled E. coli, which were opsonized with antibodies and complement factors, were incubated with leukocytes. After incubation, the increased fluorescence of the phagocytes was due to the uptake of bacteria. The fluorescence intensity is dependent on the amount of phagocytosed bacteria. A sample chilled on ice was used for control.

### Tumor necrosis factor alpha (TNF-α)

Intracellular TNF-α production in monocytes and macrophages was analyzed before (t = 0) and after 2 h, 6 h, and 24 h. For detection, an anti-human TNF-α Pycoerythrin (PE) monoclonal antibody (mAb) was applied (Pharmingen, Germany). The specificity of the antibody had been validated in blocking experiments. To 40 μl blood, 145 μl RPMI and 15 μl 40 μM Monensin were added (Monensin prevents cytokine release by blocking the golgi apparatus). Cells were incubated for 2 h at T = 37°C and 5% CO_2 _atmosphere. After washing, monocytes were surface stained by anti-human CD14 TriColor mAb (Medac, Germany). Red blood cells were lysed using FACS™ Lysing Solution (Becton Dickinson, Germany). Cells were fixed and permeabilized by addition of 250 μl Cytofix/Cytoperm™ (Becton Dickinson, Germany). Finally, the retained intracellular TNF-α was stained by the anti-TNF-α PE mAb. For a positive control, cells were stimulated with 0.25 μg Lipopolysaccharide (LPS), following the same procedure as mentioned above.

### Surface activation markers

Surface activation markers were analyzed before (t = 0) and after 2 h, 6 h, 24 h, 48 h, 72 h, and 96 h. For the detection of activation markers, anti-human CD69 PE (Holzel Diagnostika, Germany), CD25 Fluoresceinisothiocyanat (FITC), CD71 FITC, CD11b PE (Diaclone Research, Germany) and HLA-DR PerCP (Becton Dickinson, Germany) mAbs were used. For a positive control, cells were stimulated with 10 ng/ml Phorbol-12-Myristat-13-Acetat (PMA) and 1 μg/ml Ionomycin for 2 h, while macrophages/monocytes were stimulated with 1.25 μg/ml LPS for 2 h. To distinguish between leukocytes, additional surface markers, anti-human CD45 Allophycocyanine (APC), CD19 APC (Caltag Laboratories, Germany), CD3 Tri/Color (DAKO, Germany) and CD14 FITC (Diaclone Research, Germany) mAbs were used for detection. Blood samples (40 μl) were stained for 4-color flow cytometry analysis. After staining, red blood cells were lysed, subsequently leukocytes were analyzed. Antibody specificity was validated by isotype controls (Becton Dickinson, Germany).

### Specific antibody development

Serum samples were analyzed before (t = 0) and after 24 h, 48 h, 72 h, 96 h, 10 d, 12 d, and 14 d. Specific anti-LK565 antibodies were determined via indirect ELISA. Indirect ELISA was performed in a 96-well plate by LK565 immobilization overnight. Optimal concentrations had previously been determined by common cross-testing. Serum was obtained from blood samples after centrifugation at 400 G. After incubation for 2 h at room temperature, detector antibodies (Biozol Diagnostica GmbH, Germany) were added and incubated at 4°C overnight. Washing was carried out with phosphate buffered saline (PBS) and blocking buffer. For detection, alkaline phosphatase (AP)-linked goat-anti-human IgG and IgM antibodies with para-Nitrophenylphosphate (p-NPP) as substrate (Biozol Diagnostica GmbH, Germany) were used. After development, the enzyme reaction was stopped with 0.5 M NaOH and measured at λ = 405 nm. For the functional (positive) control of the indirect ELISA, total immunoglobuline was captured polyclonally. For a negative control, the serum of a person who had not been exposed to the contrast agent was obtained.

## Results

We investigated 15 volunteers in a clinical phase one trial over a period of 14 days. The kinetics of activation surface markers CD69, CD25, CD71 and CD11b on leukocytes, the phagocytosis capacity, and TNF-α production in monocytes were analyzed, as was the specific antibody production after LK565 application.

The expression of CD69 [15], CD25 [16], CD71, HLA-DR [17], and CD11b [18] is increased after activation [19]. CD69 will be presented quickly after activation on almost all leukocytes normally after some hours but on neutrophils even after a few minutes due to mobilisation of intracellular storages [20]. The α-chain of the IL-2 receptor CD25, also known as the high-affinity receptor on lymphocytes, is developed after 6 hours at the earliest and up to 1–2 days. Transferrin receptor CD71 correlates directly with cell proliferation and a late marker which can be expected after 3–4 days similar to antigen presentation via MHC-II analyzed as HLA-DR. Integrin expression (CD11b) on monocytes and neutrophils is associated with phagocyte migration into tissue [21].

Independent of dosage or repeated contact, the contrast agent LK565 was well tolerated by all volunteers. After exposure to LK565 no erythema, no drop in blood pressure, neither significant change of the heart frequency nor fever occurred (data not shown). The volunteers did not complain of relevant clinical symptoms such as chest discomfort or asthenia. It exhibited good opacification of both ventricles a few seconds after application (figure 2). While the minimum dosage of 0.15 mg/kg LK565 was sufficient for just one echocardiogram, a suitable duration of echo contrast was obtained at a 0.4 mg/kg dose. A further increase in dosage provided no improvement in quality or contrast duration (data not shown).

**Figure 4:**
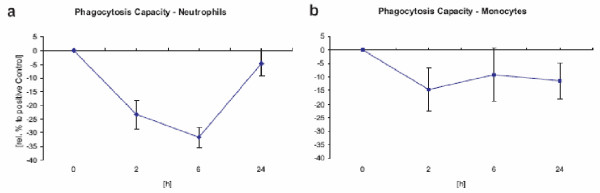
**Phagocytosis capacity after LK565 exposure **Phagocytosis capacity refer to opsonized E. coli of neutrophils (a) and monocytes (b) in vitro illustrated in percent of control after LK565 exposure (n = 15, error bars show standard error).

The uptake of LK565 led to the saturation of phagocytes. The uptake capacity of the phagocytes from blood samples 6 h after injection was exhausted (figure 4). Neither macrophages nor neutrophils phagocytosed any more labeled bacteria after LK565 uptake, the observed effect was reversible. After 24 h the phagocytic capacity reached almost the starting level.

**Figure 5:**
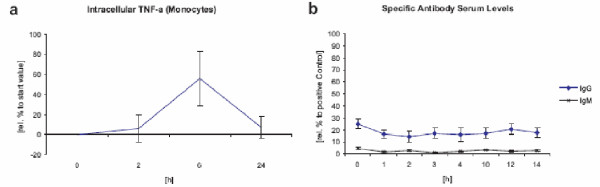
**TNF-α and specific antibody production after LK565 application **(a) intracellular TNF-α (Monocytes) after LK565 exposure relative to starting value and (b) LK565 specific antibody production (IgM, IgG) relative to positive control (n = 15, error bars show standard error).

During the first 24 h an increase in intracellular TNF-α production was detected in monocytes and macrophages (figure 5a). However, in relation to the positive control after 2 h of LPS stimulation, the slight increase in intracellular TNF-α was negligible. No connection between repeated exposure and dosage was found. The amount of intracellular TNF-α decreased to starting levels after 24 h.

**Figure 6:**
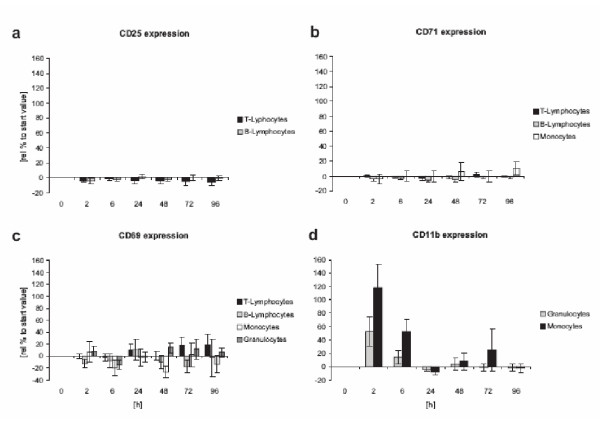
**Activation marker expression after LK565 application **CD25, CD71, CD69 and CD11b expression on different cell types after LK565 exposure relative to starting value (n = 15, error bars show standard error).

An increase in integrin expression CD11b was detected on monocytes and macrophages 6 hours after application. Compared to the positive control, the upregulation of the integrin was only poor. In all cases, the upregulation of the integrin was only of brief duration and returned to normal levels after 24 h (figure 6d). Only a slight and short increase in CD69 after 6 h was determined in neutrophils in a few cases. Most volunteers exhibited no CD69 increase in neutrophils. Even less CD69 expression was found on monocytes and macrophages where CD69 stimulation is associated with the production of prostaglandines, leukotriens and TNF-α. Only minor CD69 expression was observed on T-cells and on B-cells (figure 6c). No increase of CD25 and CD71 (also on monocytes) was detected (figure 6a,6b).

**Figure 7:**
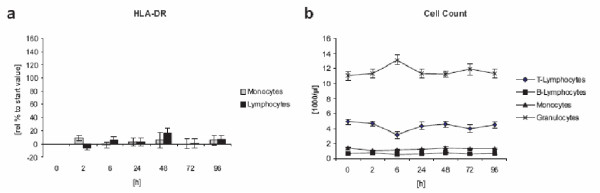
**HLA-DR expression after LK565 application **HLA-DR expression relative to starting value and differential cell count after LK565 application (n = 15, error bars show standard error).

Discrete MHC class II (HLA-DR) upregulation on macrophages and lymphocytes was observed (figure 7a). We found no further evidence of an adaptive immune response via antibody production. None of the serum samples during our clinical trial exhibited any development of specific antibodies (IgM, IgG) versus the contrast agent (figure 5b).

## Discussion

The results show that there is only a slight activation of leukocytes esp. of phagocytes after application of LK565, a particular contrast agent based on a polymer of the naturally occurring amino aspartic acid. No specific antibody development against the contrast agent was detected. Thus, the risk of a major immunological activation cascade after repeated dosage can be regarded as minor. Since LK565 does not lead to an adaptive immune activation in this study, the possibility of an overexpression of IgE, which plays a key role in allergic diseases, also becomes more unlikely. Although none of the 15 volunteers enrolled in this phase I study developed an allergic-type reaction, such reactions could, however, occur in further phases of clinical testing or application. An allergic reaction against a pharmaceutical drug is a rare event which is impossible to exclude even in large clinical studies.

An increase in CD11b/CD18 is connected with phagocytosis and the diapedesis readiness of phagocytes. LK565 is eliminated from the blood stream via phagocytosis, therefore neutrophils exhibited an increase in CD11b expression and phagocytosis activity. This is also connected to a temporary higher intracellular TNF-α level in monocytes.

On lymphocytes, especially T-cells, the cascade of CD69, CD25 and CD71 is closely related to an immune response. After expression of CD69, the interlinkage of the receptor leads to proliferation and secretion of IL-2, IFN-γ and TNF-α. After effective activation, the T-cells secrete IL-2 which activates the development of the high-affinity IL-2 receptor CD25 and therefore launches cell proliferation with an expression of CD71 after 3 – 4 days. During our study after LK565 application no major activation cascade was detected on either T-cells or B-cells.

As mentioned above, the best contrast performance for echocardiography was obtained at a LK565 dosage of 0.4 mg/kg. Opacification of both ventricles was not enhanced by a further increase in dosage. Bearing in mind the fact that the contrast agent is phagocytosed and uptake capacity is limited, it is recommended that the daily dosage does not exceed 90 mg, which is equivalent up to three echocardiograms per day.

## Conclusions

To minimize the risk of an undesirable adverse event such as an anaphylactoid reaction, immunological studies should be included in clinical trials for new UCAs. The use of LK565 as another new ultrasound contrast agent (UCA) with a comfortable duration of signal enhancement esp. in echocardiography should be encouraged as a means to provide additional diagnostic information without causing a major activation cascade or triggering an adaptive immune response. This can be an advantage for the "difficult-to-image" patient with adverse reactions related to other UCAs.

## Competing interests

H.K. Maerz received salary for 6 months from Dr. F. Koehler Chemie GmbH, Alsbach-Haehnlein, Germany, the developer/manufacturer of LK565. R. Zotz is co-author of one of several patents related to LK565 (Zotz R, Erbel R, Krone V, Magerstädt M, Walch A (1992): Ultrasonic contrast agents, processes for their preparation and the use thereof as diagnostic and therapeutic agents. United States Patent 5.137.928).

## Authors' contributions

BF: Prepared and edited the manuscript as well as the figures und performed echocardiographic evaluations; HKM: Drafted the manuscript and participated in the design of the study esp. immunological aspects; SO: Carried out TNF-α evaluations, recruited and coordinated the clinical management of the patients; SP: Carried out expression pattern analysis, recruited and coordinated the clinical management of the patient; IL: Participated in the study design; US: Reviewed the manuscript according to study design and immunological aspects PW and TG: Reviewed the manuscript and edited the figures; RZ: Participated in the development of LK565, the study design and coordination. All authors read and approved the final manuscript.

## References

[B1] Feinstein SB (1992). Myocardial perfusion: contrast echocardiography perspectives. Am J Cardiol.

[B2] Meltzer RS, Tickner EG, Sahines TP, Popp R (1980). The source of ultrasound contrast effect. J Clin Ultrasound.

[B3] Cheng SC, Dy TC, Feinstein SB (1998). Contrast echocardiography: review and future directions. Am J Cardiol.

[B4] Smith MD, Kwan OL, Reiser HJ, DeMaria AN (1984). Superior intensity and reproducibility of SHU-454, a new right heart contrast agent. J Am Coll Cardiol.

[B5] Keller MW, Glasheen W, Kaul S (1989). Albunex: a safe and effective commercially produced agent for myocardial contrast echocardiography. J Am Soc Echocardiogr.

[B6] Zotz RJ, Genth S, Erbel R, Dieterich HA, Meyer J (1994). Contrast echocardiography of the left ventricle an independent predictor of pulmonary artery pressure?. Int J Card Imaging.

[B7] Schneider M, Arditi M, Barrau MB, Brochot J, Broillet A, Ventrone R, Yan F (1995). BR1: a new ultrasonographic contrast agent based on sulfur hexafluoridefilled microbubbles. Invest Radiol.

[B8] Grayburn P (1997). Perflenapent emulsion (EchoGen): a long-acting phase shift agent for contrast echocardiography. Clin Cardiol.

[B9] Cohen JL, Cheirif J, Segar DS, Gillam LID, Gottdiener JS, Hausnerova E, Bruns DE (1998). Improved left ventricular endocardial border delineation and opacification with OPTISON (FS069), a new echocardiographic contrast agent. Results of a phase-III multicenter trial. J Am Coll Cardiol.

[B10] Zotz R, Genth S, Grande J, Walch A, Ziehn P, Krone V, Schuler G (1996). Polymer microparticles for right and left heart echocardiography and imaging myocardial perfusion after peripheral vein injection. Z Kardiol.

[B11] Zotz RJ, Schenk S, Kuhn A, Schlunken S, Krone V, Bruns W, Genth S, Schuler G (2001). Safety and efficacy of LK565 – a new polymer ultrasound contrast medium. Z Kardiol.

[B12] Maerz HK, Okorokow S, Polata S, Lehmann 1, Schneider P, Zotz R (1999). The Fate of ECHO Beads 11 – Immunological Response or Not?. Immunbiol.

[B13] Morrissette N, Gold E, Aderem A (1999). The macrophage – a cell for all seasons. Trends Biol.

[B14] Hofsli E, Bakke 0, Nonstad U, Espevik T (1989). A flow cytometric and immunofluorescence microscopic study of tumor necrosis factor production and localization in human monocytes. Cell Immunol.

[B15] Testi R, D'Ambrosio D, De Maria R, Santoni A (1994). The CD69 receptor: a multipurpose cell-surface trigger for hematopoietic cells. Immunol Today.

[B16] Werfel T, Boeker M, Kapp A (1997). Rapid expression of the CD69 antigen on T cells and Natural Killer cells upon antigenic stimulation of peripheral blood mononuclear cell suspensions. Allergy.

[B17] Arva E, Andersson B (1999). Kinetics of cytokine release and expression of lymphocyte cell-surface activation markers after in vitro stimulation of human peripheral blood mononuclear cells with Streptococcus pneumoniae. Scand J Immunol.

[B18] Prieto J, Beatty PG, Clark EA, Patarroyo M (1988). Molecule mediating adhesion of T and B cells, monocytes and granulocytes to vascular endothelial cells. Immunology.

[B19] Thompson HL, Matsushima K (1992). Human polymorphonuclear leukocytes stimulated by tumour necrosis factor-alpha show increased adherence to extracellular matrix proteins which is mediated via the CD11b 18 complex. Clin Exp Immunol.

[B20] Craston R, Koh M, McDermott A, Ray N, Prentice HG, Lowdell MW (1997). Temporal dynamics of CD69 expression on lymphoid cells. J Immunol Meth.

[B21] Lundgren-Akerlund E, Olofsson AM, Berger E, Arfors KE (1993). CD11b/CD18-dependent polymorphnuclear leukocyte interaction with matrix proteins in adhesion and migration. Scand J Immunol.

